# A Tailor-Made, Mirror-Based Infrared Scanner for the Reflectography of Paintings: Development, Features, and Applications

**DOI:** 10.3390/s23094322

**Published:** 2023-04-27

**Authors:** Marco Gargano, Daniele Viganò, Tiziana Cavaleri, Francesco Cavaliere, Nicola Ludwig, Federica Pozzi

**Affiliations:** 1 Dipartimento di Fisica Aldo Pontremoli, Università degli Studi di Milano, Via Celoria 16, 20133 Milano, Italy; marco.gargano@unimi.it (M.G.); daniele.vigano@mi.infn.it (D.V.); francesco.cavaliere@fisica.unimi.it (F.C.); nicola.ludwig@unimi.it (N.L.); 2 Istituto Nazionale di Fisica Nucleare (INFN), Sezione di Milano, Via Celoria 16, 20133 Milano, Italy; 3 Centro per la Conservazione ed il Restauro dei Beni Culturali “La Venaria Reale”, Via XX Settembre 18, 10078 Venaria Reale, Italy; federica.pozzi@ccrvenaria.it; 4 Dipartimento di Economia, Ingegneria, Società e Impresa (DEIM), Università della Tuscia, Via del Paradiso 47, 01100 Viterbo, Italy

**Keywords:** infrared reflectography, spherical scanning, SWIR scanner, underdrawings, InGaAs camera, high-resolution imaging, painting investigation

## Abstract

Since infrared reflectography was first applied in the 1960s to visualize the underdrawings of ancient paintings, several devices and scanning techniques were successfully proposed both as prototypes and commercial instruments. In fact, because of the sensors’ small dimension, typically ranging from 0.1 to 0.3 megapixels, scanning is always required. Point, line, and image scanners are all viable options to obtain an infrared image of the painting with adequate spatial resolution. This paper presents a newly developed, tailormade scanning system based on an InGaAs camera equipped with a catadioptric long-focus lens in a fixed position, enabling all movements to occur by means of a rotating mirror and precision step motors. Given the specific design of this system, as the mirror rotates, refocus of the lens is necessary and it is made possible by an autofocus system involving a laser distance meter and a motorized lens. The system proved to be lightweight, low cost, easily portable, and suitable for the examination of large-scale painting surfaces by providing high-resolution reflectograms. Furthermore, high-resolution images at different wavelengths can be obtained using band-pass filters. The in-situ analysis of a 16th-century panel painting is also discussed as a representative case study to demonstrate the effectiveness and reliability of the system described herein.

## 1. Introduction

In the study of paintings, it is well known that most paint layers based on modern or ancient pigments are transparent to near-infrared radiation between 0.8 and 2.5 microns [1,2,3,4,5,6]. Because of this phenomenon, hidden features related to the lowermost layers of panel or canvas paintings, such as underdrawings or compositional changes made by the artist himself or within later interventions, can be revealed through technical analysis by means of infrared reflectography (IRR) [7,8,9,10]. In addition, increased transparency in the near-infrared range is also the basis of many techniques that are able to increase the legibility of text in ancient or degraded documents. A combination of the increased transparency of pigments and the opacity of carbon-based drawings provides sufficient contrast for the latter to be efficiently visualized by detectors in the near-infrared band. 

The most widely used devices employ silicon semiconductors, indium gallium arsenide (InGaAs), mercury cadmium telluride (HgCdTe, MCT), germanium (Ge), or platinum silicide (PtSi). Silicon detectors are the most widespread and inexpensive, albeit only sensitive up to 1000 nm [3], which yields acceptable results under various measurement conditions. However, detectors for cultural heritage applications are typically based on InGaAs cameras, cooled and uncooled, due to their high signal-to-noise ratio and sensitivity between 900 and 1700 nm. A modified version of these detectors, equipped with a suitable cooling system, enables extended sensitivity up to 2500 nm. Despite providing good sensitivity in the reflectographic infrared range, Ge-, MCT-, and PtSi-based detectors are very expensive. The main limitation shown by silicon detectors is related to the opacity of certain pigments in the band where this material is most sensitive: for instance, copper-based pigments such as malachite and azurite, as well as some dark-tone earths, are not transparent when examined up to the operating band of silicon detectors. Recently, detectors working in the mid-wave and long-wave infrared were proposed for specific applications with promising results [11,12,13,14,15,16]. In some cases, however, scanning is required due to the very low pixel size of short-wave infrared (SWIR) detectors (640 × 520 pixels for the largest ones). This ensures image collection with an adequate spatial resolution to enable in-depth studies of a work’s painting or drawing techniques—including close examination of thin or subtle brushstrokes, shadings, and hatching with different thickness and saturation, as well as decorations with extremely fine details.

To date, there exists a number of scanning systems, both commercially available and tailormade prototypes. Each of these have advantages and drawbacks in terms of cost, scanning area, scan time, and spectral resolution. Scanning can rely on single-pixel systems [17,18] or image plane systems [18,19,20,21], some operating with a multispectral approach [4,21,22,23,24,25] and others integrating 3D information [26]. The instrument presented here, of the second type, was developed as the evolution of a former prototype with the specific aim to address a series of observed issues [27]. In this earlier version, image acquisition relied on the movement of the entire camera, including the optics, as a focusing system prompted its translation back and forth to maintain a certain distance from the painting examined when the pan and tilt position changed. Travel of the linear stage was limited to 10 cm, which restricted the possibility of focusing and thus the painting’s total scanning area. In addition to a longer acquisition time, the focusing system relied on theoretical values calculated based on the initial distance from the painting and subsequent pan and tilt position. The new instrument proposed herein allows us to improve the scanning method by increasing the area to be scanned as well as the focus accuracy, while decreasing the acquisition time by moving a mirror instead of the camera.

In this context, this paper describes the operating principle, construction process, and testing of a device that is capable of collecting high-resolution reflectographic images based on spherical scanning, a well-known technique in photographic applications [28]. In this system, image acquisition is performed by rotating the camera around its nonparallax point [29], allowing the collection of a series of images that are then merged to expand the final field of view.

Several products are currently used to obtain panoramic images: these include, among others, tripod heads that enable rotations around one or two axes and, in some configurations, are available with a motorized option. However, these heads are primarily designed for photographic cameras and lack important features such as full remote control, sufficient angular resolution, and, most importantly, the ability to implement autofocus for proper image focusing. Our tailormade device was developed and upgraded to meet all the requirements related to resolution, field of view, versatility, and cost. The prototype proposed in this work is based on an InGaAs camera mounted in a fixed position and pointed at a 45° elliptical mirror that is moved to scan the target surface. Rotation of the mirror, instead of the camera, allows for faster and more precise movements with less inertia due to the reduced weight of the mirror compared to that of the camera. Focusing is ensured by a motorized lens, which, again, promotes rapid and accurate focusing. The system is controlled in the LabVIEW™ environment using inexpensive, but high-performance, Arduino boards and modules, with a wireless connection.

After a series of preliminary tests, the system was evaluated for the in-situ technical analysis of a 16th-century panel painting. The artwork is currently undergoing scientific analysis and conservation treatment at the Centro per la Conservazione ed il Restauro dei Beni Culturali (CCR) “La Venaria Reale”, located in Venaria Reale, province of Turin, Italy.

## 2. Materials and Methods

### 2.1. Optical and Mechanical Components

**Figure 1:**
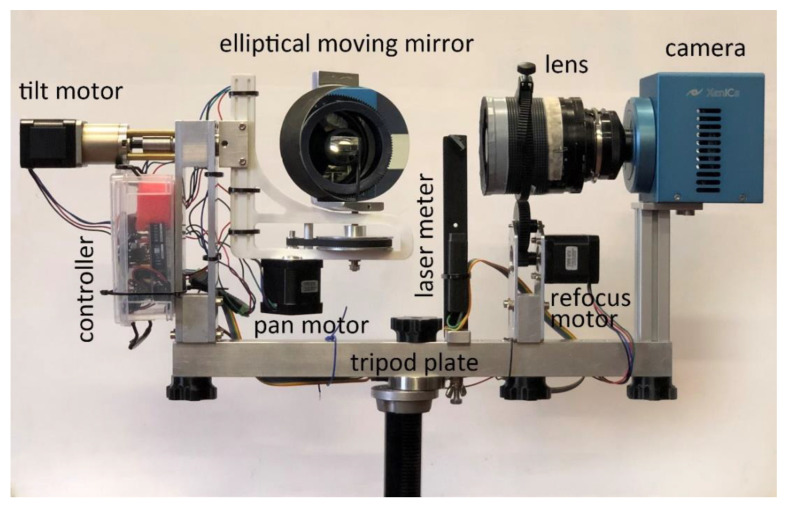
Components and modules of the scanning system.

The prototype proposed in this article consists of a modular system that can be further implemented and upgraded. As illustrated in Figure 1, its main components are the camera and lens, a mirror, three motors for movement, and a laser for distance measurement. Once assembled, the system can be attached to a supporting bar, which is then mounted on a tripod stand. Figure 1 shows the system fully mounted and ready for scanning. The basic idea is to equip the scanning system with a focusing module consisting of a time-of-flight distance meter and a motor acting on the focus ring of the lens. Focusing is a crucial step for the optical configuration of the system: in fact, in order to reduce the scanner’s weight and size without unduly decreasing the dimension of the scanned area, linear translations must be avoided in favor of a spherical system, as commonly employed in panoramic photography.

However, the typical situation in panoramic photography is significantly different from the reflectographic setup. As a matter of fact, in the former case, objects are normally located far away from the camera, while usual distances between IRR systems and target paintings range between 30 and 200 cm. Under these conditions, a panoramic scan would produce increasing deformations as the angle of rotation from the normal to the painting’s surface increases. In addition, moving away from the normal would result in a noticeable loss in terms of spatial resolution. These issues can be addressed by increasing the painting–camera working distance through the use of a lens with a long focal length so as not to decrease spatial resolution. The limited depth of field of lenses with long focal lengths, however, makes refocusing necessary. Details of the components used to assemble the scanning prototype are reported in Table 1.

### 2.2. Electronic and Software Components

**Figure 2:**
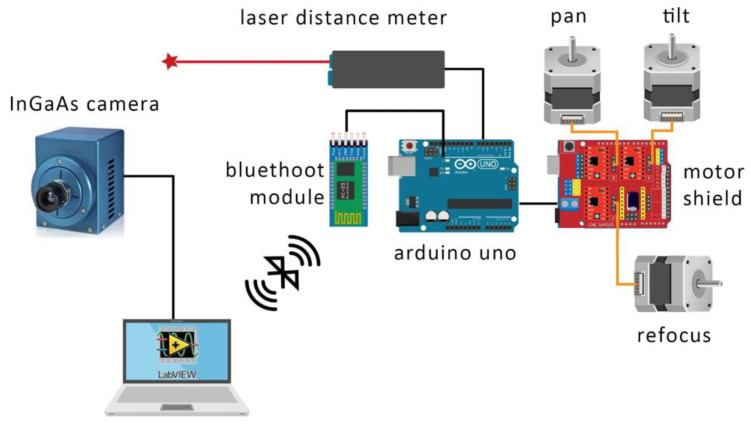
Graphical scheme showing the assembling of the individual components into the operating scanning system.

As shown in Figure 2, the camera and modules are controlled in the LabVIEW™ environment, using an Arduino Uno microcontroller equipped with a motor shield board and a Bluetooth Serial Port Protocol module. This configuration allowed the research team to reduce the number of wired connections to one only (for the camera), while operating all other controls wirelessly.

**Figure 3:**
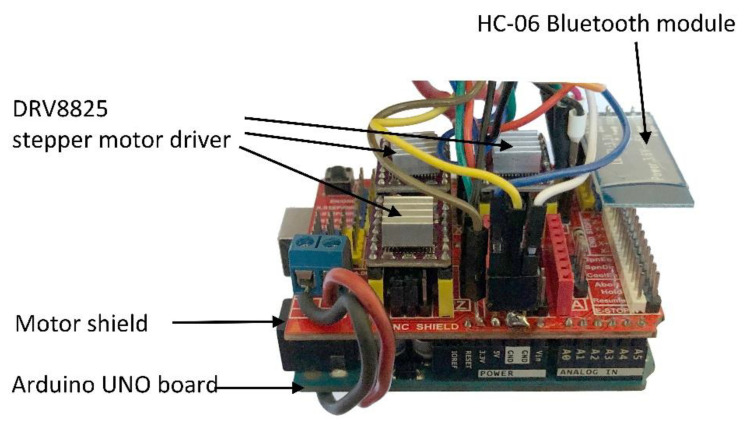
Control module of the scanning system based on the Arduino Uno board.

Figure 3 illustrates the control module used for the scanning system, which is based on an Arduino Uno board, a motor shield, three stepper motor drivers, and a Bluetooth communication module.

**Figure 4:**
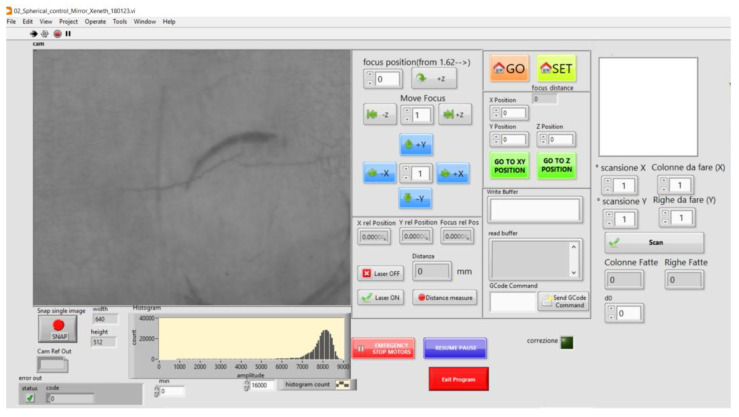
Front panel view of the application developed in the LabVIEW™ environment.

The screen interface used to control the software program can be seen in Figure 4. In the LabVIEW™ environment, it was possible to integrate the motion control, refocusing system, and distance measurement within a single application that allows the operator to manage both the software and hardware components.

### 2.3. Optical Scheme

**Figure 5:**
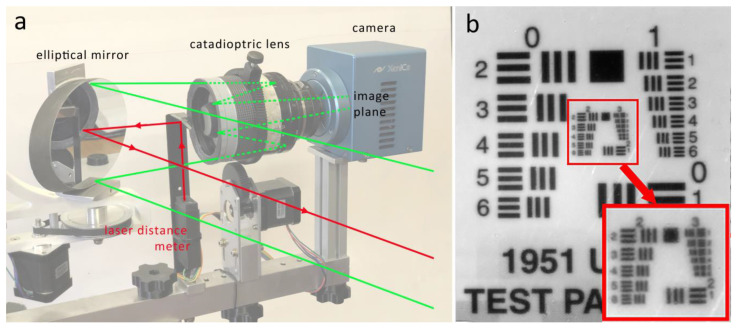
(**a**) Scheme of the system’s optical design showing the path of the laser beam used to measure the camera–painting working distance (red lines) and the path of the infrared radiation focused on the camera plane for image acquisition (green solid lines for the path outside the lens and green dashed lines for the path inside the lens). (**b**) USAF resolution target as captured by the scanning system.

The system’s innovative optical design is based on a mirror that is moved instead of the camera, yielding a reduction of one fifth in weight and enabling lens and camera to be varied without significantly changing the system configuration. The catadioptric lens, with a central obstruction that contains the secondary mirror, lends itself very well to be used in combination with a time-of-flight laser distance meter. It is possible, in fact, to frontally accommodate an additional small mirror at a 45° angle to reflect the laser beam toward the elliptical mirror and then to the painting surface for the distance measurement, as the mirror position changes and so does the area of the painting viewed by the camera. A scheme of the system’s optical design is shown in Figure 5a. The laser beam used to measure the camera–painting working distance first hits the 45° mirror and then the elliptical mirror in the optical path (red lines); after distance measurement, the laser is disabled and the infrared radiation is focused on the camera plane for image acquisition (green lines). Given the peculiarity of this optical scheme, the scanning area is limited by two factors: first, the lens’ field of view is partially obscured by the mirror, and second, its shallow depth of field prevents the ability to image areas that are located far from the normal to the painting surface. In this configuration, maximum dimension of the scanning area is 2 × 2 m, a limit that can be exceeded by increasing the lens’ focal length and moving away from the painting, and performing focus stacking for more peripheral areas. Additionally, the system can be equipped with band-pass filters, mounted between the camera and lens, to carry out a multiband acquisition. Lens focus was calibrated against the stepper motors’ position using a U.S. Air Force (USAF) resolution optical target, which also allowed us to evaluate the instrument’s actual spatial resolution, usually reported as a theoretical value dividing the sensor size by the field of view in real-world units. Figure 5b shows the optical target located at a distance of 1.7 m, as in the actual capture of the painting. The system is able to resolve up to group 2 and element 6 on the select target, corresponding to 7.13 line/pairs per millimeter and equal to a resolving power of 70.15 μm, which indicates the approximate resolution limit.

**Figure 6:**
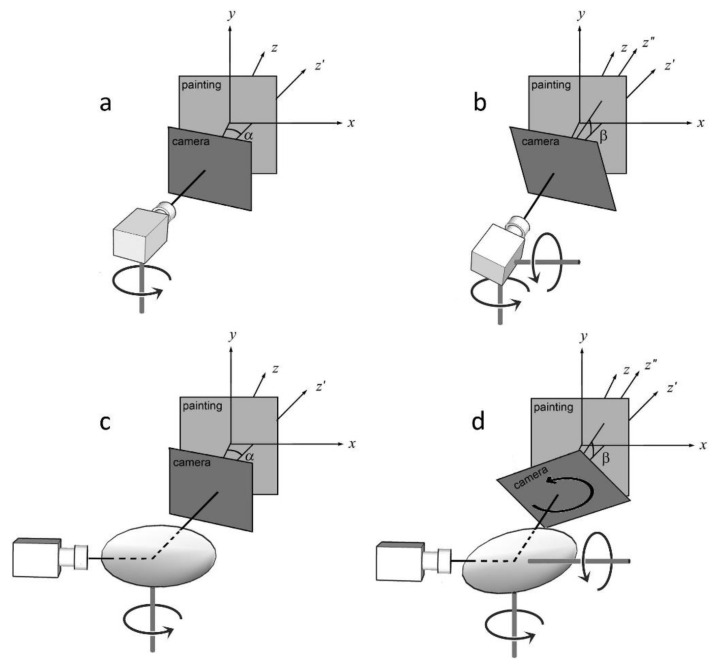
Rotation of the camera coordinates (*x*, *y*, *z*) of an angle *α* and to the equivalent reference frame (*x*′, *y*′, *z*′) for camera (**a**) and mirror (**c**) movement. If we add a rotation *β*, the new reference frames (*x*″, *y*″, *z*″) for the camera (**b**) and mirror (**d**) are different since the reference frame for the mirror undergoes an additional rotation *β* around the *z*″ axis with respect to the camera.

Images obtained by scanning, in which the mirror is rotated and tilted as in the proposed case, turn out to be rotated around the optical axis due to the mirror’s rotation with respect to the lens’ optical axis. As illustrated in Figure 6, a reference frame $\left(x^{\prime },y^{\prime },z^{\prime }\right)$ can be obtained by considering the painting coordinates $\left(x,y,z\right)$ with the camera placed orthogonally to its surface and then applying a rotation by an angle α. Figure 6a,c, representing rotation for the camera and mirror, show no difference in this reference frame. On the other hand, rotation of the camera or mirror by an angle β yields a new reference frame $\left(x^{\prime \prime },y^{\prime \prime },z^{\prime \prime }\right)$ that appears different for each configuration. The reference frame represented in Figure 6c, in fact, undergoes an additional rotation β around the $z^{\prime \prime }$ axis with respect to the camera alone (Figure 6b).

### 2.4. Post-Processing and Merging

A challenging phase in the creation of a high-resolution reflectogram is the post-processing of an enormous amount of single 14-bit grayscale captured images. The additional rotation of each image further increases the level of difficulty that burdens the merging process. After detector calibration, accomplished through the Xeneth software wizard, dark subtraction and flat field correction are needed in the capture conditions. The so-obtained images can be corrected through the imtransform function in MATLAB Processing Toolbox™ [30], using the angular position information embedded in each recorded image. Subsequent merging can be achieved by means of several methods available for this specific purpose. The most commonly used applications in panoramic photography are PTGui 12.21 [31], Huging 2022.0.0 [32], GigaPan Stitch 2.1.0161 [33], Image Composite Editor 2.0.3 [34], the merging tool of Adobe Photoshop^®^ 24.3.0 [35], and other computational methods based on different approaches [36]. In this research, all the abovementioned applications were tested to select the most efficient, which turned out to be Image Composite Editor: this application was able to combine all 2000 images collected from the top and bottom sections of the painting by using the panorama stitching option with known image positions. Distortions were found to be quite small and were adjusted in Adobe Photoshop^®^ [35], using the painting’s visible light photograph as a reference.

### 2.5. Case Study

**Figure 7:**
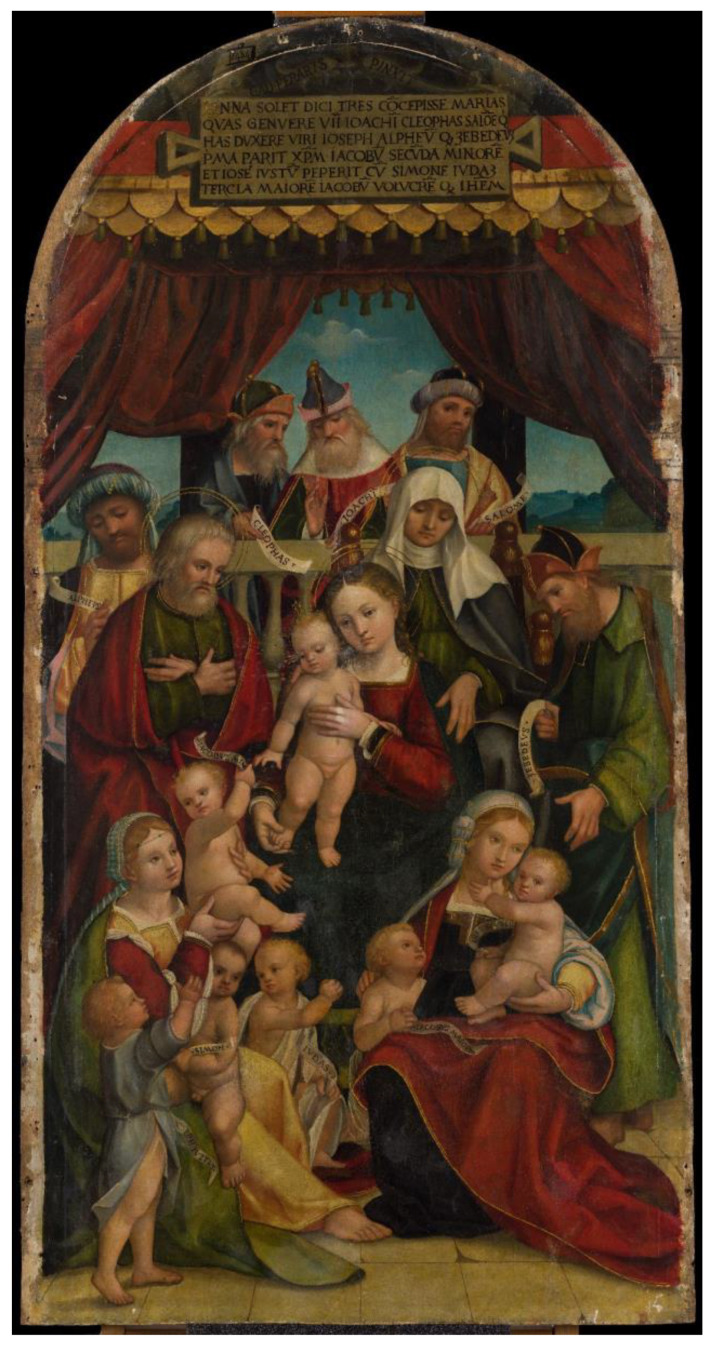
Gandolfino da Roreto (attributed), *Genealogy of the Virgin*, ca. 1510–1520, painting on wooden panel, 173 × 83 cm. Church of Santa Maria Assunta, Grignasco (Novara), Italy.

**Figure 8:**
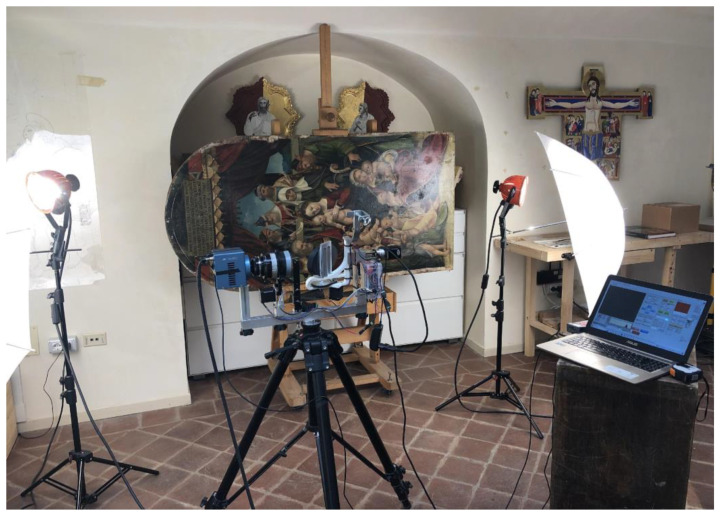
Instrumental setup for IRR of the panel painting in the CCR “La Venaria Reale” conservation laboratories. Scanning was performed by placing the work in horizontal position on an easel.

After a series of preliminary tests, the infrared scanning system described herein was evaluated for the in-situ technical analysis of a remarkable 16th-century panel painting, titled *Genealogy of the Virgin* and attributed to Italian painter Gandolfino da Roreto, who was active in the Piedmont region during the Early Renaissance (Figure 7). The painting, owned by the Diocese of Novara, comes from the Church of Santa Maria Assunta in Grignasco, province of Novara, Italy, and is approximately dated to 1510–1520. Dimensions of the panel are 173 × 83 cm. Over the centuries, the painting has undergone at least one significant conservation treatment. IRR was performed as part of a broader analytical campaign aiming to shed light on the work’s materials and techniques, as well as any extant issues possibly related to previous interventions or to its conservation condition deteriorating over time. The painting is currently held in the CCR “La Venaria Reale” laboratories, where it is undergoing scientific analysis and conservation treatment under the supervision of the Soprintendenza Archeologia, Belle Arti e Paesaggio per le Province di Biella, Novara, Verbano-Cusio-Ossola e Vercelli. The instrumental setup for IRR is illustrated in Figure 8; scanning was performed by mounting the painting on a supporting easel in horizontal position and positioning the scanner 150 cm away from the painting surface. Two 350 W halogen lamps and diffusers were used for lighting.

## 3. Results and Discussion

**Figure 9:**
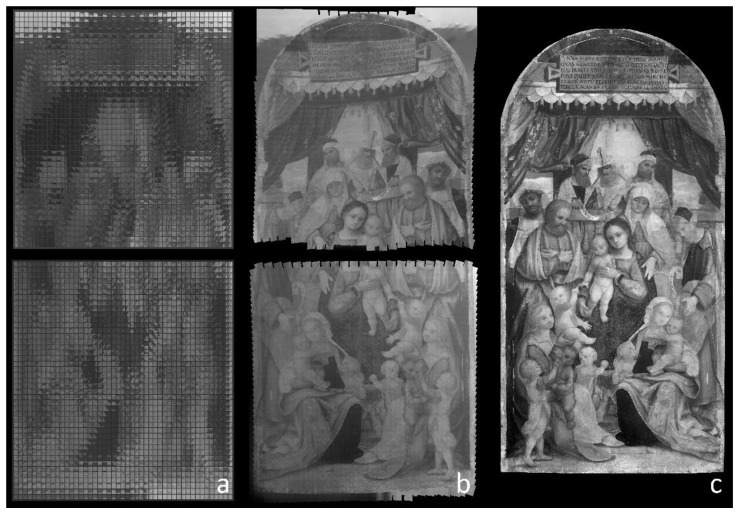
(**a**) All individual infrared images captured from the top and bottom sections of the panel painting are placed side by side in preparation for the merging process. (**b**) Stitching of the individual 2000 + 2000 image sets. (**c**) Final recomposition with flat field correction, image registration, and gray levels optimization.

Reflectographic analysis of the panel was performed in two separate scans, due to the large surface of the painting examined, in order to minimize the blur effect caused by the lens’ limited depth of focus. All acquisitions were carried out in the 1.0–1.7 μm spectral range. A critical parameter that affects the scan time significantly is the choice of overlapping area between adjacent images: while with camera rotation this can equal 20–30%, with mirror rotation it is recommended not to fall below 50%, which also facilitates the automatic stitching process that follows. For the panel painting investigated in this paper, a total of 4000 individual images were captured: 2000 for the top half and 2000 for the bottom half (Figure 9a). This choice was meant to ensure uniform lighting over the entire painting surface and to limit the distortion of peripheral areas, while facilitating image recomposition. Total acquisition time was about 2 h. Although the focusing method employed proved to be fast, in the presence of very dark backgrounds the measurement often gave rise to errors. This encountered problem was solved by imposing a condition on the distance measurement, according to which if the latter differed by more than 1% from a theoretical value, calculated assuming that the painting was perfectly flat, the measured value would be discarded in favor of the theoretical value.

The best-suited stitching software to manage the significant number of images acquired automatically was found to be Image Composite Editor. In fact, this application was able to fully assemble the 2000 images collected from the artwork’s top and bottom sections with limited resulting distortions, as shown in Figure 9b. The final IRR image, adjusted using the painting’s visible light photograph as a reference, is shown in Figure 9c. Hugin is also potentially performant, but in many cases requires the manual input of control points in order to perform the alignment. In fact, the image rotation effect caused by the mirror’s tilt movement greatly burdens the recognition of individual images in the alignment process. Preliminary correction of the images, achieved by straightening the individual shots for the mirror position angle, did not produce a significant gain in terms of processing time. 

**Figure 10:**
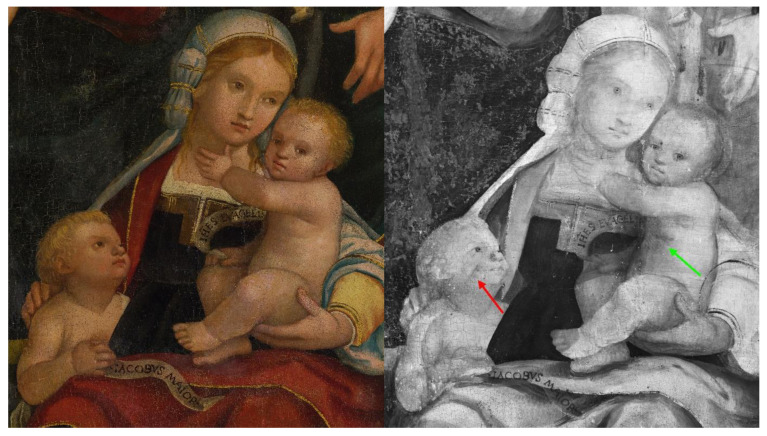
Detail of the painting’s lower right quadrant, depicting the Virgin’s sister, Salome, with her two sons, James the Greater and John the Evangelist. Compared to the visible light photograph (**left**), the IRR image (**right**) shows compositional changes such as the shading on John the Evangelist’s chest and an overall modified position for James the Greater, highlighted with green and red arrows.

**Figure 11:**
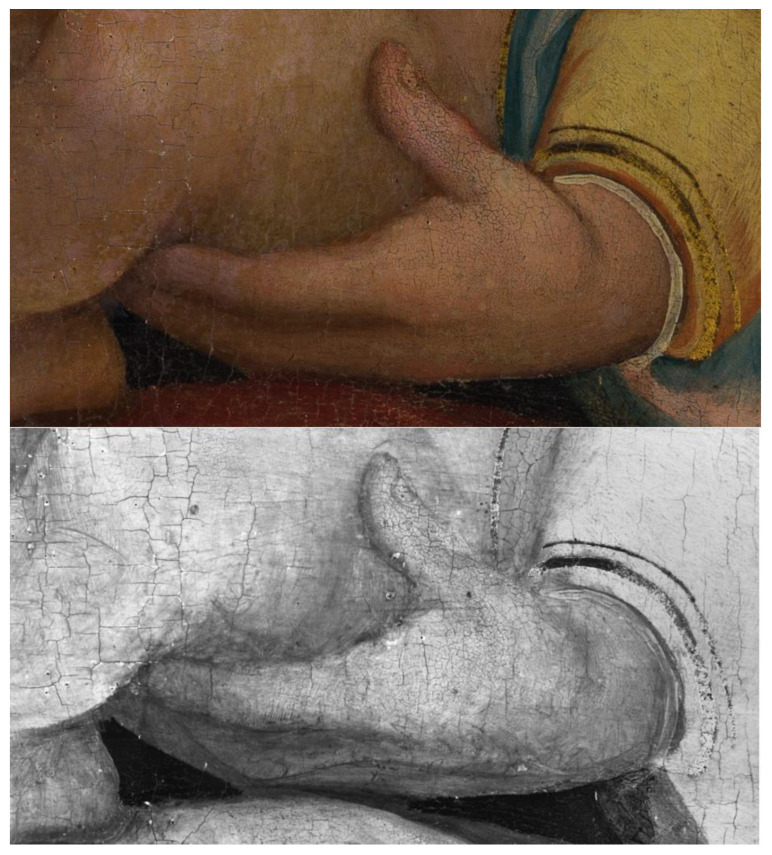
Detail of Salome’s proper left hand holding the baby. Compared to the visible light photograph (**top**), the IRR image (**bottom**) shows compositional changes such as the figure’s middle finger in bent position.

The reflectogram reveals interesting aspects of the artist’s technique. Most significantly, there is evidence of the presence of an underdrawing over the entire surface of the panel painting. Such underlying drawing is executed with a brush and a carbon-based ink, likely used in combination with a dry medium to adjust or reinforce certain details in the composition (Figure 10 and Figure 11). Figure 10 depicts a detail of the Virgin’s sister, Salome, with her two sons, James the Greater and John the Evangelist: in this area, the outline strokes of the drawing and the shading on John the Evangelist’s chest are noticeable (highlighted with a green arrow). On the other hand, a distinct trace along James’ proper right arm, of difficult interpretation, might be cautiously attributed to a *pentimento* or compositional change, with the child formerly depicted closer to Salome and one of his eyes visible in IRR where the cheek is currently located (highlighted with a red arrow). An additional change between subsequent versions of the painting is detected in correspondence to Salome’s proper left hand, whose middle finger in the underdrawing appears bent (Figure 11).

**Figure 12:**
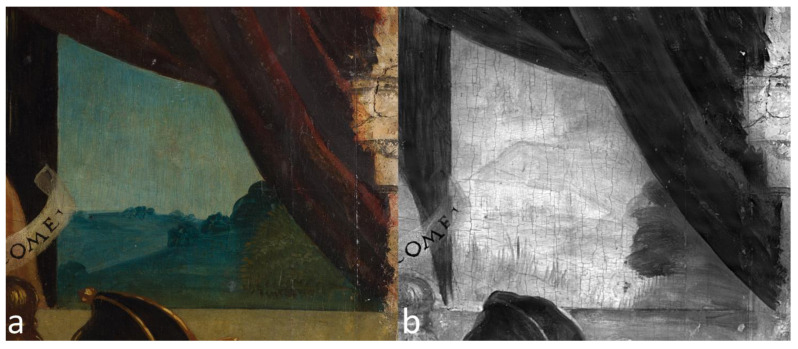
Detail of the painting’s upper right quadrant, depicting a landscape scene beyond a curtain. Compared to the visible light photograph (**a**), the IRR image (**b**) shows compositional changes such as a house with roof, walls, and windows.

Furthermore, an outstanding compositional change appears to have been made in the background landscape seen beyond a curtain in the painting’s upper right quadrant (Figure 12). Indeed, a house with roof, walls, and windows, present in the underdrawing, was apparently not reported in the final painting phase, which includes, instead, a natural landscape with hills and vegetation. The corresponding area in the painting’s upper left quadrant does not show any compositional changes in the infrared reflectogram. 

**Figure 13:**
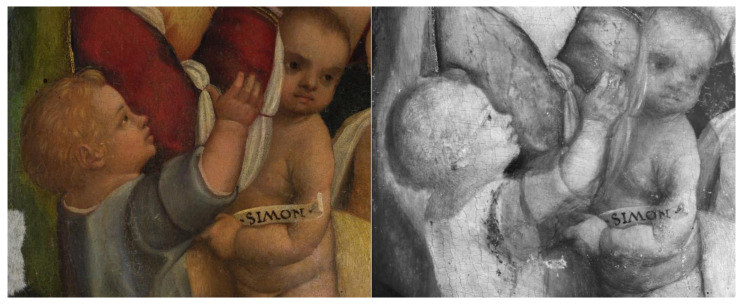
Detail of the painting’s lower left quadrant, depicting two children. Compared to the visible light photograph (**left**), the IRR image (**right**) shows compositional changes such as a subsequent addition of a sleeve in Joseph’s robe.

Further fascinating details of the artist’s technique can be drawn from close inspection of the area in Figure 13. In fact, the sleeve of Joseph’s robe appears to have been added at a later stage, on top of the figure’s bare arm; careful shading on the elbow and armpit is also visible. This observation, along with the infrared response of Joseph’s back, suggests that the child was originally naked, similar to all others in the composition, and clothing was later added likely because he is located in the foreground.

## 4. Conclusions

The spherical infrared scanning system implemented in this work, equipped with mirror handling, was proven to provide a conveniently portable and versatile system for reflectographic applications even on large-scale panel paintings. The use of a mirror significantly reduces the weight of the handling system. On the other hand, the addition of an autofocus mechanism on the lens, on a theoretical level, enables the operators to have virtually no limits in terms of selected dimensions of the scanned surface. In fact, the main drawback of this system is related to the lens’ depth of field and the partial obstruction of the lens’ field of view due to the mirror. The combination of the LabVIEW™ software with the low-cost, modular Arduino system enables the creation of an inexpensive and wireless handling and control system.

The system is modular and it is possible to change the type of camera and lens; as an alternative to the laser focusing system, a theoretical focusing system based on the angular position of the framed area can be used. In-situ testing on Gandolfino da Roreto’s 16th-century large-scale panel painting made it possible to evaluate the effectiveness and performance of the proposed system. The selected Image Composite Editor stitching software was able to stitch a considerable number of images with adequate overlapping. Further developments are currently underway to improve acquisition times and preprocess the collected images in order to facilitate merging.

## Figures and Tables

**Table 1:** Details of the components used to assemble the scanning prototype.

Component	Manufacturer	Model	Tech Specifics
Camera	Xenics™, Leuven, Belgium	Xeva-1.7-320 InGaAs camera	Sensitivity between 0.9 and 1.7 µm, 320 × 256-element array with a pixel pitch of 20 µm, and cooling system, yielding 14-bit grayscale images.
Lens	Tamron™, Saitama, Japan	500 mm f/8 SP macro-tele lens	Compact design considering its focal length; it has few glass elements and a minimum focal distance of approximately 1.7 m. At this distance, the optical magnification is about 3:1, which means that at a 1.7 m distance a given painting would be sampled at 560 pixels per inch at the center of the scanned area.
Mirror	GSO™, Guan Sheng Optical, Taiwan	Elliptical mirror	The mirror surface has aluminate layer providing 94% reflectivity, mirror size 104 × 150 mm, precision 1/12 RMS, thickness 18.7 mm.
Distance meter	Chengdu JRT Meter Technology Co., Ltd, Chengdu, China	Time-of-flight laser distance meter	Wavelength 635 nm, range 0.02–50 m, accuracy ±2 mm, power < 1 mW (class II laser). Distance is measured by measuring the time taken by the laser beam to travel a distance. Module is controlled by using a TTL/serial communication protocol.
Motors	OSM Technology Co., Ltd., Nanjing, China	Stepper motors	Current 2A, torque 59 Ncm, each motor has different gear ratio depending on the weight to be moved. Angular resolution of all movements is 0.02°.

## Data Availability

All data generated during this study are either included in this published article or available from the corresponding author upon reasonable request.
